# Immune Response, Inflammation, and the Clinical Spectrum of COVID-19

**DOI:** 10.3389/fimmu.2020.01441

**Published:** 2020-06-16

**Authors:** Luis F. García

**Affiliations:** Grupo de Inmunología Celular e Inmunogenética, Facultad de Medicina, Sede de Investigación Universitaria, Universidad de Antioquia, Medellín, Colombia

**Keywords:** COVID-19, SARS-CoV-2, immune response, T cells, antibodies, cytokines, inflammation, spectrum

## Abstract

The current COVID-19 pandemic began in December 2019 in Wuhan (China) and rapidly extended to become a global sanitary and economic emergency. Its etiological agent is the coronavirus SARS-CoV-2. COVID-19 presents a wide spectrum of clinical manifestations, which ranges from an asymptomatic infection to a severe pneumonia accompanied by multisystemic failure that can lead to a patient's death. The immune response to SARS-CoV-2 is known to involve all the components of the immune system that together appear responsible for viral elimination and recovery from the infection. Nonetheless, such immune responses are implicated in the disease's progression to a more severe and lethal process. This review describes the general aspects of both COVID-19 and its etiological agent SARS-CoV-2, stressing the similarities with other severe coronavirus infections, such as SARS and MERS, but more importantly, pointing toward the evidence supporting the hypothesis that the clinical spectrum of COVID-19 is a consequence of the corresponding variable spectrum of the immune responses to the virus. The critical point where progression of the disease ensues appears to center on loss of the immune regulation between protective and altered responses due to exacerbation of the inflammatory components. Finally, it appears possible to delineate certain major challenges deserving of exhaustive investigation to further understand COVID-19 immunopathogenesis, thus helping to design more effective diagnostic, therapeutic, and prophylactic strategies.

## Introduction

The current COVID-19 pandemic, caused by the coronavirus SARS-CoV-2 and initiated with the first cases observed in Wuhan (China) in December 2019, has expanded dramatically throughout the world (1, 2). This expansion has had devastating effects in many countries due to its contagiousness and the high number of patients presenting with severe infections and elevated death risk, requiring specialized medical care in intensive care units (ICU). For this reason, the WHO declared it a Global Sanitary Emergency on January 30, 2020 (3). An important aspect to highlight during the present crisis is the speed at which research studies have been developed, leading toward a better understanding of the epidemiology, clinical manifestations, risk factors, and transmission dynamics (1, 4–8), as well as to the identification of the etiological agent (9, 10), including its genome, morphological structure, and molecules (11–13), its relationship with other coronaviruses (14), its entrance into the host cells by binding the Angiotensin II Converting Enzyme (ACE2) (9), its intracellular replication (15), and the immune response of the infected individuals (16–19). All these studies aim at developing diagnostic tests, strategies for clinical management, effective antiviral agents, and eventually, production of a protective vaccine.

The goal of this review is to analyze the main aspects of the immune response against SARS-CoV-2 and the relationship between the protective and inflammatory responses and COVID-19 clinical spectrum, ranging from asymptomatic to severe clinical presentations. The review also highlights the principal immunological research challenges posed by COVID-19 pandemics. The immune response in humans and experimental animals against infection by SARS-CoV and MERS-CoV has been studied extensively and there are many excellent reviews (20–23). However, due to the similarities of COVID-19 with SARS and MERS, it will be necessary, at certain specific points, to cite the research done on those infections.

## The Virus

The virus responsible for the epidemic that began in Wuhan was simultaneously identified by Wu et al. (9), and by Zhou et al. (10), who named it WH-Human 1 and 2019-nCoV, respectively. These researchers also deciphered the virus genome, its origin from bat coronaviruses, and ACE2 as its receptor on the membrane of host cells. On February 11, 2020, the WHO officially named the infection COVID-19 and the virus as SARS-CoV-2 (24).

SARS-CoV-2 belongs to the family Coronaviridae, which includes a large number of species capable of infecting various wild animals, some of which also affect humans (25–27). In humans, most coronavirus infections result in mild respiratory Infections and may be responsible for 20–30% of common colds (28). However, both SARS-CoV and MERS-CoV, which emerged in the last two decades, were responsible for epidemics of severe respiratory syndromes. The three coronaviruses causing more serious pathologies belong to beta-CoV (23) and, despite their genomic and structural similarities, they differ significantly epidemiologically. SARS-CoV and MERS-CoV have a low transmissibility but a high lethality, while SARS-CoV-2 has an extremely high transmissibility and a degree of lethality not yet established globally.

Coronaviruses have a single-stranded positive RNA of nearly 30 Kbp, a spheroidal shape, and a diameter of 80–120 nm. Their envelope contains the spike –S-, membrane -M-, and envelope -E-, proteins, and the nucleocapsid -N- inside the virion that covers the RNA (23). On the genome, from 5′ to 3′, are located the genes for the replicases ORF *1a,b* which occupy two thirds of the genome and code for the polyproteins pp1a and pp1b (9, 23, 29). Located toward the 3′ end are the genes for structural proteins *S, E, M*, and *N* (9, 23, 30).

Protein *S* is the best studied of the coronaviruses proteins, since it contains the Receptor-Binding-Domain (RBD) for the ligand on the host cell membrane, and also has epitopes recognized by T and B cells, which induce the production of neutralizing antibodies (31). *S* is a type I trimeric glycoprotein that protrudes from the virion membrane, giving it the appearance of a crown. *S* is formed by two subunits: S1, or bulb, that contains the RBD (32–39); and S2, or stalk, responsible for the fusion of the virion with the host cell membrane (23, 35, 36, 38, 40–42).

As described above, the main receptor for SARS-CoV and SARS-CoV-2 on the membrane of the target cells is the Angiotensin 2 Converting Enzyme (ACE2), a metallopeptidase present on the membrane of many cells, including type-I and -II pneumocytes, small intestine enterocytes, kidney proximal tubules cells, the endothelial cells of arteries and veins, and the arterial smooth muscle, among other tissues (43, 44). RBD-ACE2 binding induces conformational changes on *S* that lead to cleavage of S1 and S2, a process mediated by the serine protease TMPRSS2, allowing S2 to facilitate the fusion of the virus envelope with the cell membrane, thus permitting viral RNA entrance into the cytoplasm of the target cells (23, 31, 35, 42, 45). Thereafter, viral RNA serves as a template for the translation of the polyproteins pp1a and pp1b that are cleaved into 5–16 non-structural proteins (nsp2-nsp9), which in turn induce rearrangement of the membranes to form the vesicles where viral replication and transcription complexes are anchored. The virions are assembled in the ER-Golgi and mature virions are subsequently released by the secretory pathway (23).

## The Infection

The COVID-19 pathological process exhibits a wide spectrum of clinical manifestations, ranging from asymptomatic infections, to mild (common cold-type), moderate, and finally severe (~15%) infections; the latter frequently requires hospitalization in ICU to ensure assisted respiratory support and other medical treatments until recovery, or possibly death, of the patient. The wide spectrum of clinical manifestations found in COVID-19 patients has been associated with risk factors such as gender and age. Diabetes, cardiovascular disease, or diseases, or treatments affecting the immune system result in the highest risk of severe disease and death (6, 8, 8, 46–48). It is, however, estimated that nearly 80% of all infections remain undocumented, either because patients are asymptomatic or present with very mild symptoms (49). From the epidemiological point of view, these inapparently infected persons may have low viral loads, while still disseminating the virus and can therefore be responsible either for silent epidemics, leading to infection in more susceptible people who will eventually develop a clinical disease, or for contributing to the establishment of herd immunity (28, 50).

SARS-CoV-2 is acquired by exposure to microdroplets present in the exhalates of infected individuals or by contact with viral particles present in contaminated fomites. Once the virus reaches the bronchioles and alveolar spaces, the main targets are the cells of the bronchial epithelium and the type-II ACE2^+^ pneumocytes of the alveolar epithelium. SARS-CoV infection induces autophagy (51, 52), detachment of the basal membrane, and inhibition of ACE2 expression (20, 53), hence allowing angiotensin II to bind the AT1aR receptor, resulting in acute lung damage (54). Importantly, the main early defense mechanism of the infected cell is the production of type-I and type -III IFN and, although coronaviruses are sensitive to their anti-viral effects, they are able to inhibit its induction (16, 20, 52, 55). The release of large number of virions leads to both infection of neighboring target cells and viremia, the latter resulting in systemic infection since ACE2^+^ cells are widely distributed in many tissues (43, 44).

## The Innate Immune Response

During viral infections, after viruses enter the host cells they are recognized by Pattern Recognition Receptors (PRR) such as TLR7 and TLR8 in the case of single-stranded RNA viruses, RIG-I-like (RLRs), and NLR, all expressed by epithelial cells as well as by local cells of the innate immune response, such as alveolar macrophages (23). Upon ligand binding, PRRs recruit adaptor proteins which activate crucial down-stream transcription factors, including interferon regulatory factor (IRF), NF-κB, and AP-1, resulting in production of the Type-I and -III antiviral Interferons and different chemokines (56). These chemokines attract more innate response cells [polymorphonuclear leukocytes, monocytes, NK cells, dendritic cells (DC)], which also produce chemokines, such as MIG, IP-10, and MCP-1, capable of recruiting lymphocytes, which in turn, will recognize the viral antigens presented by DCs (20, 22). Recent publications highlight the initial phases of the SARS-CoV-2 infection, compared to other coronavirus, and their effects on subsequent immune and inflammatory responses. Chu et al. (57) compared the *in vitro* infection of human lung explants with SARS-CoV and SARS-CoV-2 and demonstrated that both viruses can equally infect type-I and -II pneumocytes, plus alveolar macrophages, although SARS-CoV2 had a better capacity to replicate in pulmonary tissues. Interestingly, while SARS-CoV induced the expression of IFN-I, IFN-II, and IFN-III, SARS-CoV-2 failed to induce any such immune mediators and was also less efficient in inducting other cytokines. SARS-CoV induced the production of the 11 cytokines studied, while SARS-CoV-2 induced only five (IL-6, MCP1, CXCL1, CXCL5, and CXCL10/IP10). Blanco-Melo et al. (55) studied the transcriptional response to SARS-CoV-2, compared to SAR-CoV, MERS-CoV, respiratory syncytial virus (RSV), parainfluenza virus 3 (HPIV3), and influenza A virus (IAV), in *in vitro* infection of respiratory cell lines, experimental *in vivo* infection of ferrets, and post-mortem lung samples of COVID-19 patients. Their results show that SARS-CoV-2 induces a particular signature characterized by reduced IFN-I and IFN-III responses and significant induction of multiple proinflammatory chemokines, IL-1B, IL-6, TNF, and IL1RA. These findings were further supported by the increased serum levels of these molecules in COVID-19 patients. Altogether, these reports strongly suggest that SARS-CoV-2 differs from other coronaviruses in its capacity to replicate within the pulmonary tissue, elude from the antiviral effects of IFN-I and IFN-III, activate innate responses, and induce the production of the cytokines required for the recruitment of adaptive immunity cells.

## Adaptive Immune Response

The transition between innate and adaptive immune responses is critical for the clinical progress of SARS-CoV-2 infection. It is at this crucial moment when immune regulatory events, still poorly understood, will lead to the development of either a protective immune response or an exacerbated inflammatory response (18, 19, 58, 59). The protective response is T cell dependent, with CD4 helping B cells, geared toward the production of specific neutralizing antibodies, and cytotoxic CD8 cells capable of eliminating infected cells. It is worth noting that 80% of the infiltrating cells in COVID-19 are CD8 (16). Contrariwise, a dysfunctional response, unable to inhibit viral replication and elimination of the infected cells, may result in an exacerbated inflammatory response leading possibly to a cytokine storm, manifested clinically by severe acute respiratory distress syndrome (ARDS) and systemic consequences, such as disseminated intravascular coagulation. In a SARS-CoV primate model of infection, Clay et al. (60) showed that the virus replicated in the lungs until Day 10 post-infection; but, surprisingly, lung inflammation was more intense after virus clearance, reaching its peak at Day 14 and remaining so until Day 28. These results suggest that an early phase dependent on virus replication does occur, while a later viral-independent, immune-dependent phase seems to be accompanied by an exacerbated inflammatory component. The viral-independent phase has been explained by the inflammatory reaction secondary to ACE2 inhibition or by an autoimmune phenomenon due to the epitope spreading caused by prolonged tissue destruction (20, 61). It remains to be demonstrated whether a similar two-phase course also occurs in COVID-19.

Although T and B cells, macrophages, and DCs do not express ACE2, some reports suggest that DC-SIGN may serve as a *trans* receptor for SARS-CoV on DCs, which even when not infected may transfer the virus to other susceptible cells (22, 23, 62). Recently, Vandakari and Wilce (63) reported that CD26, an aminopeptidase involved in T cell activation, may bind to the *S* protein of SARS-CoV-2, resulting in a non-productive T cell infection; Wang et al. (64) reported that CD147, a protein of the immunoglobulins superfamily that induces the metalloproteinases of the extracellular matrix, binds to the S1 domain and facilitates viral entrance into host cells. The significance of non-productive T cells infection is not clear; however, it is tempting to speculate that it may be related to the lymphopenia found in patients with SARS, MERS, and COVID-19 (65). The binding of SARS-CoV-2 S protein to molecules like CD26 and CD147, which participate in T cell activation, would suggest that a non-productive T cell infection may result in activation-induced cell death (AICD). MERS-CoV has been reported to induce T cells apoptosis (23, 66), and there is evidence that T cells are functionally exhausted in patients with severe COVID-19 (67).

## The Antibody Response

Multiple evidences support that the humoral response, mainly antibodies against the *S* protein, blocks virus attachment to susceptible ACE2^+^ cells (33, 41, 68, 69). However, there are still many questions regarding the significance of antibodies against the different viral proteins, and the cross reactivity of antibodies against other highly prevalent alpha- and beta-coronavirus, although it seems that cross reactivity occurs mostly within the beta-coronaviridae (61, 70), particularly between SARS-CoV and SARS-CoV-2 that share 90% of the amino acid sequence in S1 (31). However, it can also happen with other antigens, as demonstrated in the outbreak of HCoV-OC43 in British Columbia (Canada) where cross reactivity of anti-N antibodies with SARS-CoV was found (71). In this respect, it is interesting that there is no information regarding whether survivors of the SARS and MERS epidemics became infected with SARS-CoV-2, and if so, the nature of their clinical and immunological behavior.

IgM and IgA antibodies can be detected early during the 1st week of symptom onset, whereas IgG can be detected at around 14 days after the initiation of symptoms (61, 70, 72); however, given the short time elapsed since the beginning of the COVID-19 pandemic, it is not known how long the protecting levels of these blocking antibodies will remain active. Nevertheless, in a cohort of SARS survivors followed for 6 years, Tang et al. (73) found that anti-SARS-CoV antibodies were undetectable in 21/23 patients and that none of them had specific memory B cells, whereas specific memory T cells were present in 14/23 (60.9%) of the SARS survivors studied. Although the diagnostic value of the serological tests for COVID-19 is not yet fully defined (70, 74–76), it should be stated that the study of the antibodies against different SARS-CoV-2 antigens, in different populations and at various times during the pandemic, would be an important way of understanding the dynamics of transmission and seroprevalence as a proxy to herd immunity. Furthermore, it is equally important to conduct serial antibody titers measurements in cohorts of COVID-19 survivors in order to determine how long the immune memory remains active and its effect on the possible reemergence of SARS-CoV-2, or other coronavirus outbreaks.

### The Role of Secretory Immunoglobulin a (sIgA)

It is worth noting that the role of secretory immunoglobulin A (sIgA) in COVID-19 has received little attention, despite the fact that SARS-CoV-2 enters the body through the respiratory mucosa and sIgA is fundamental to the mucosal defenses. Furthermore, several studies into COVID-19 have shown the presence of serum IgA against SARS-CoV-2 (70, 77–79) and, in preclinical studies with anti-SARS vaccines, administered either sub-lingually or intranasally, the presence of neutralizing IgA was demonstrated in bronchoalveolar lavages (80–82). These findings support the importance of investigating the presence of sIgA in secretions of patients with COVID-19 and defining its possible anti-viral neutralizing activity in respiratory tract mucosa (83).

### Antibody-Dependent Enhancement (ADE)

An intriguing phenomenon that worries many clinicians and researchers is the “Antibody-Dependent Enhancement” (ADE), which could be linked to the severity of coronavirus infections and could possibly create difficulties with new vaccines (84–86). Ho et al. (87) studying the antibody response in SARS, found that patients with more severe clinical courses had earlier and higher antibody responses, and hypothesized that earlier responders may have had, during the acute phase, cross-reacting antibodies with non-SARS coronaviruses. Jaume et al. (88) and Yip et al. (89) demonstrated that anti-S antibodies, while inhibiting viral entrance in permissive cells, potentiated the infection by binding to IgG Fc receptor-II positive (FcγRII^+^) cells, like B cells and macrophages. Thus, IgG anti-S antibodies bound to FcγRII on mononuclear phagocyte membranes enhance viral entrance through canonical viral-receptor pathways, as recently shown for MERS-CoV (90), thereby activating these cells and inducing the production of proinflammatory cytokines.

## The Clinical-Immunogical Spectrum of COVID-19

In order to understand COVID-19 immunopathogenesis, it is important to elucidate what lies at the root of immune response failure occurring in infected individuals resulting at times in deviation of the protective response into a dysfunctional program, leading to cytokine release syndrome (CRS) with severe inflammation and, eventually, a multi-systemic failure. A better understanding of these events would contribute to the design of differential therapeutic approaches, depending on the stage of the disease, and to the delineation of prognostic, and predictive biomarkers. Unfortunately, there are no studies on the immune response in infected asymptomatic individuals, which would allow a better characterization of the protective immune response as it occurs under the natural conditions of the infection process. Thus, the present view is based on the comparison between patients with moderate and those with severe infections, and also with those in the convalescent stage. Another aspect to be explored is the effect of previous exposure to other less virulent coronaviruses that may have cross-reactivity with more virulent ones. Additionally, most of the studies have been done using blood samples, which do not necessarily correlate with the events going on in the affected tissues. Fortunately, several studies on bronchoalveolar lavage cells were published recently, as will be discussed below.

**Figure 1:**
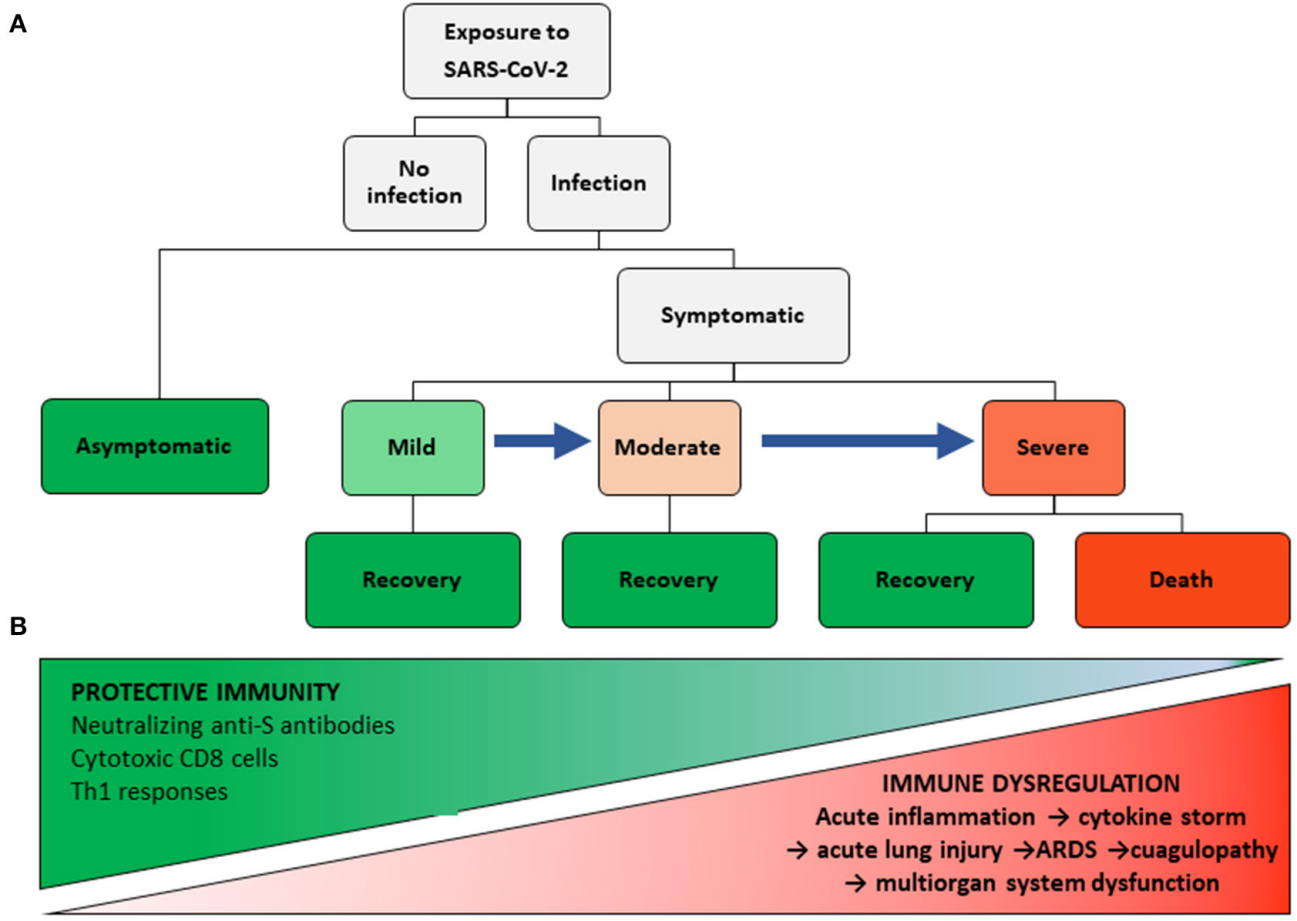
COVID-19 Clinical and immunological spectra. **(A)** Clinical stages of COVID-19. **(B)** Protective immunity and inflammatory spectra.

From an immunological point of view, the wide clinical spectrum of COVID-19 allows us to postulate different hypotheses, some of them which have already been proven, the remaining requiring more information and longer follow-up observation of recovered patients. Figure 1A shows diverse outcomes during the course of COVID-19 and allows for an analysis of the immune response at each clinical stage. However, it must be noted that the immune response is conditioned by epidemiological variables, such as intensity and duration of exposure to the virus and possible variations in viral virulence and, on the host side, genetic susceptibility/resistance and health conditions at the time of exposure. The latter includes, among other variables, age and the existence of comorbidities that may directly affect the immune system (8, 48).

Despite its high infectivity, not everyone exposed to SARS-CoV-2 becomes infected (15). The reasons for such resistance are still unknown. It is possible that a small, occasional inoculum does not reach the lower respiratory tract, where susceptible target cells are found. Nevertheless, as yet unidentified genetic conditions may also explain this *per se* resistance. On this regard, no association of SARS with ACE2 polymorphisms was found (91).

A central tenet of our view on COVID-19 immunopathogenesis is that a protective immune response must be present in patients with asymptomatic and mild infections, and even in some with moderate infections who do not progress to severe disease. This response must be capable of inhibiting viral replication and eliminating the host's infected cells with minimal tissue damage and low inflammatory manifestations (Figure 1B). The adaptive response includes the existence of genetic conditions for viral antigen presentation by HLA-I and II molecules to CD8 and CD4 T cells, respectively. In this context, Grifoni et al. (92), using a bioinformatic approach, identified 241 candidate epitopes for HLA-II alleles in SARS-CoV-2, and 628 for class I alleles, which may be bound by the more common HLA alleles, irrespective of their ethnic group. The high number of epitopes, also present in SARS-CoV (93), may explain the lack of a consistent association of SARS with HLA antigens (94–98).

## Histopathology

In patients with COVID-19, the *post mortem* histopathological findings are similar to those reported in SARS (99) and MERS (100). Xu et al. (101) described bilateral diffuse alveolar damage (DAD) with cellular fibromyxoid exudates, pneumocytes desquamation and hyaline membrane formation, multinucleated syncytial cells, atypical enlarged pneumocytes with large nuclei, amphophilic granular cytoplasm, and prominent nucleoli, as well as interstitial mononuclear infiltration. Zhang et al. (47) also reported DAD with denudation of the alveolar epithelia, reactive type-II pneumocyte hyperplasia, intra-alveolar fibrinous exudate, and loose fibrous plugs, along with loose interstitial fibrosis and chronic inflammatory infiltrate. Also, these authors, using an anti-Rp3 NP of SARS-CoV-2, demonstrated the presence of the virus on alveolar epithelial cells, including damaged, desquamated cells within the alveolar space, but its presence was only minimally detected on the blood vessels and the interstitium. Taken together, the histopathological findings in COVID-19 fatalities support that, in addition to the direct cytopathic effect of SARS-CoV-2 on the pneumocytes, an immunological response exists that includes a severe inflammatory reaction and extensive lung damage (22).

## The Immunological Profile of COVID-19 Patients

There is consensus that in severe COVID-19 infection, an exacerbated pulmonary and systemic inflammatory response occurs, with increased serum levels of inflammatory markers, such as C-reactive protein (CRP), lactic dehydrogenase (LDH), ferritin, D-dimer, and IL-6 (2, 5, 6, 67, 102), all of which may result in cytokine storm (102–104), similarly to SARS and MERS (20, 105). Table 1 compares the blood immunological profile of patients with moderate and severe COVID-19.

**Table 1:** Clue immunological findings in blood of patients with moderate or severe COVID-19.

**References**	**COVID-19 moderate**	**COVID-19 severe**
Zhou et al. (48)	No data	**↓** Lymphocytes, **↓**CD4
Huang et al. (5)	**↑**PMNs, ↓ Lymphocytes	**↑** IL-2, IL-7, IP10, MIP1A, TNF
Wu et al. (8)	**↑** PMNs, **↓** Lymphocytes, **↓**CD4,	**↑** IL-6 (at risk of death)
Qin et al. (106)	No data	**↑** PMNs, **↓** Lymphocytes, **↓**T (Th1 y Tregs), B, NK; **↑** T “naïve,” **↓**T memory **↑** IL-2R, IL-6, IL-8, IL-10
Chen et al. (17)	**↓** Lymphocytes **↑**IL-2R, IL-6, IL-10, TNF; **→**IL-1β,IL-8	**↓↓** Lymphocytes, **↓**CD4 and CD8 **↑↑**IL-2R, IL-6, IL-10.TNF
Wan et al. (110)	**↓** CD4, CD8, B, NK **→**IL-4, IL-10, IL-17, TNF, IFN;**↑**IL-6	**↓↓** CD4, CD8, B, NK **↑↑**IL-6
Zheng et al. (67)	**↓** CD8, NK (NKG2^+^CD107a^+^IFN-γ^+^grzB^+^)	**↓↓** CD8, NK (NKG2^+^CD107a^+^IFN-γ^+^grzB^+^)
Lei et al. (107)	**↓**NK, **↑**Tγδ CD25^+^, **→**PD-1	
Zhou et al. (108)	**↓** Lymphocytes, **↓** Monocytes, **↓**CD4; **→**PMNs, B, NK; **↑**Tim3^+^Pd-1^+^ **↑** MonocytesCD14^+^CD16^+^	**↓↓** Lymphocytes, **↓↓** Monocytes, **↓**CD4 (**↑**CD69^+^CD38^+^CD44^+^), **↓**CD8; **↑↑**Tim3^+^Pd-1^+^; **↑**CD8^+^IFN-γ^+^GM-CSF^+^ **↑↑** MonocytesCD14^+^CD16^+^
Xu et al. (101)		**↑↑**CD4+HLADR+; **↑↑**CD8+CD38+; **↑**↑CD4^+^CCR6^+^Th17
Bordoni et al. (109)	**↓** Lymphocytes, **↓** CD3, ↑ MDSC ↑ IL-1b, IL-6, Il-8,TNF	**↓** Lymphocytes, **↓** CD3, ↑ MDSC, **↓** NKperf^+^ ↑ IL-1b, IL-6, Il-8,TNF

*→, normal values; ↓, decreased, ↓↓, severe decreased; ↑, increased, ↑↑, severe increased*.

### Changes in Circulating Cells

Regarding cellular changes, most studies show that lymphopenia, although present in moderate infections, is more pronounced in severe COVID-19 (17, 108) and affects mainly T cells, including CD4 Th1 and Tregs, but particularly CD8 (17, 48, 108–110). Also, in severe COVID-19 the number of circulating naive T cells increases and the number of memory T cells decreases (106). Circulating CD8 in patients with severe COVID 19 exhibited phenotypes associated with abnormal functionality (CD8^+^IFN-γ^+^GM-CSF^+^) and exhaustion (Tim3^+^Pd-1^+^) (108) or (NKG2^+^CD107a^+^IFN-γ^+^grzB^+^) (67). The latter phenotype is also found in NK cells. Interestingly, a negative correlation has been reported between serum levels of IL-6 and IL-8 and the perforin content of NK and CD8^+^ cells, which also negatively correlate with the increased number of circulating myeloid-derived suppressor cells (MDSC) (109). Although the number of CD4 cells decreased, they expressed activation markers such as CD69, CD38, CD44, and HLA-DR, including Th17 CD4^+^CCR6^+^ cells, (108). NK cells also decreased in both moderate and severe cases of the disease (107, 110). Monocytopenia is also found in COVID-19 patients, particularly in severe cases, but the circulating monocytes belong mainly to the CD14^+^CD16^+^ inflammatory monocyte subset (108).

### Changes in Cytokine/Chemokine Plasma Levels

Plasma levels of cytokines and chemokines are also increased in COVID-19, but are higher in severe infections, and includes IL-2, IL-2R, IL-6, IL-7, IL-8 IL-10, IP10, MIP1A, and TNF-α (5, 17, 106, 109, 110). High levels of plasmatic IL-6 have been consistently reported in COVID-19 and even appear to be associated with poor prognosis and risk of death (8). Thus, its measurement has been proposed as a good biomarker to monitor these patients. Liu et al. (111) studied sixty COVID-19 patients, half of whom had a severe case of the disease and high IL-6 levels. Baseline IL-6 was higher in more severe cases and correlated with bilateral interstitial lung involvement and high body temperature, as well as with other serum markers for acute inflammation. Of the 30 patients with severe disease, 25 improved clinically and showed a significant decrease in IL-6 levels, while these levels increased in three patients with disease progression. Coomes et al. (112) performed a meta-analysis of 16 papers, that included 10,798 Chinese patients, in order to test the evidence that IL-6 levels correlate with COVID-19 severity, and the effectiveness of treatment with Tocilizumab, a humanized monoclonal antibody against IL-6 receptor. All COVID-19 patients had increased levels of serum IL-6, but it was 2.9-fold higher in patients with severe COVID-19. Twenty-one patients treated with tocilizumab improved clinically with no adverse effects or deaths. Also, Xu et al. (113) reported very promising results using Tocilizumab treatment in 20 patients with severe COVID-19; all patients improved remarkably within a few days and all were discharged from the ICU within an average of 15 days.

### Dynamics of the Immune and Inflammatory Responses

During the course of COVID-19 infection, viral replication, immune response, and inflammatory reaction are dynamic events that can change rapidly, resulting in different outcomes; several reports have addressed these changes. Thevarajan et al. (114) reported the case of a patient with mild to moderate infection that was clinically, virologically, and immunologically followed over the course of the disease, including her recovery 13 days after the initiation of symptoms, and through to Day 20 at which point she had recovered. The virus was detected on Days 4 and 5 via nasopharyngeal swabs but was undetectable thereafter. IgM and IgG anti-SARS-CoV-2 antibodies progressively increase from Day 7 through to Day 20. Circulating antibody-secreting B cells, CD3^−^CD19^+^CD27^hi^CD38^hi^, appeared in the blood at the time of viral clearance (Day 7), peaked on Day 8, and remained high through to Day 20. Follicular helper T cells (T_FH_), CD4^+^CXCR5^+^ICOS^+^PD-1^+^, were also detected on Day 7 and continued increasing through to Day 20. Activated cytotoxic CD8 T cells, CD8^+^CD38^+^HLA-DR^+^, were also present on Day 7, increased through to Day 9, and then decreased through to Day 20, although with values higher than in healthy controls. There was no increase in inflammatory CD14^+^CD16^+^ monocytes, nor in activated NK CD3^−^CD56^+^HLA-DR^+^ cells. Regarding serum cytokines, of the 17 pro-inflammatory cytokines studied, only low levels of MCP1/CCL2 were found on Days 7–9. This case is interesting since there are very few studies on patients with mild infections and because IgM and IgG antibodies, antibody secreting B cells, CD4 T_FH_ cells, and activated cytotoxic CD8 cells were shown to be circulating before resolution of the symptoms.

Ong et al. (115) compared the blood transcriptional profile of three patients in early phases of Infection -one of whom evolved to a severe disease- with 10 healthy volunteers. The main findings in the patient who progressed to severe disease was that only IL-1A and IL-1B preceded the nadir of the respiratory function, and that the expression of most inflammatory genes, particularly IL-6, IL-2, TNF-α, and IFNA1/13, peaked thereafter. Also, in this patient, transcripts associated with HLA, CD4, and CD8 T cell activation were diminished, while in the other two patients, who did not progress to severe disease, the transcription profile was comparable to that of healthy controls. The authors suggest that in the first case the decreased T cell activation may have helped the inflammatory response by the IL-1 pathway, while in the other two cases the low inflammatory response allowed a moderate T cell response.

### Effect of Age

One of the risk factors most strongly associated with severe COVID-19 and death is advanced age. Immunosenescence present in the elderly affects innate immunity (116), but mainly T cell-dependent adaptive responses (117–120). In addition, experimental evidence suggests that elderly mice have increased levels of proinflammatory cytokines and that their alveolar macrophages are refractory to activation by IFN-γ (121). This finding is relevant since the protective response that eliminates the virus depends on cytotoxic CD8 cells and Th1 responses, with IFN-γ playing an important role in both responses, as demonstrated in SARS and MERS (122, 123).

Increased susceptibility in the elderly to present with severe COVID-19 forms contrasts with the lower frequency of these forms in children and young adults. Ludvigsson (124) reviewed 45 publications on COVID-19 and found that 1–5% of the patients are children who, although they present with fever and respiratory symptoms, experience milder symptoms and among whom death was extremely rare. The increase in inflammatory markers and lymphocytopenia were also less common in children. Brodin (125) postulated the following three explanations for the milder COVID-19 presentation in children:

1 The immune response is qualitatively different in children and adults, something that has been extensively studied (126);
2 The simultaneous presence of other viruses in the mucosa of the respiratory tract, common in children, could limit the growth of SARS-CoV-2 by direct virus-to-virus competition;
3 The treatment with ACE2 inhibitors and angiotensin receptors blockers, a common procedure in hypertensive adults, up-regulates ACE2 expression, increasing susceptibility to SARS-CoV-2 infection. These theoretical possibilities require clinical and experimental validation.

## Studies in Bronchoalveolar Lavage Fluid (BALF)

Findings in blood do not necessarily explain the events occurring in tissues directly affected by the infection, thus studies in bronchoalveolar lavage fluids (BALF) are very relevant (Table 2). Xiong et al. (127) used RNA-seq to study BALFs and peripheral blood mononuclear cells (PBMC) transcriptome from three COVID-19 patients (unfortunately their clinical conditions were not reported) and from three healthy subjects. The BALF cells in these patients expressed 9,609 genes, 679 of which were up-regulated and 325 down-regulated, as opposed to controls. In PBMC, 15,726 genes were expressed, with 707 up-regulated and 316 down-regulated. BALF cells from patients showed a differential expression of genes related to viral invasion and replication (viral RNA was detected in BALFs of all three patients) such as membrane-associated proteins, endoplasmic reticulum, and viral transcription. In contrast, PBMCs showed increased expression of genes related to complement activation, immunoglobulins, and B cell-mediated responses, while some genes corresponded to the acute inflammatory response. The down-regulated genes in patients' BALF were mostly related to activation of the immune response. Comparison of the cytokine genes showed that in BALFs the genes for IL-10, CCL2/MCP-1 (together with its CCR2 receptor), CXCL10/IP-10, CCL3/MIP-1A (together with its CCR5 receptor), and CCL4/MIP1B were differentially up-regulated. Another relevant finding was that in PBMC, genes related to autophagy, apotopsis, and p53 pathways were up-regulated, a finding that could be related to the lymphopenia detected in the three patients. Interestingly, IL-6 transcripts were not increased in PBMCs, although the patients had high plasma levels of such cytokine, suggesting that circulating IL-6 could have been produced in the lungs, either by alveolar epithelial cells or by recruited inflammatory cells.

**Table 2:** Differentially expressed genes (DEGs) up-regulated in bronchoalveolar lavage fluid (BALF) of patients with moderate or severe COVID-19.

**References**	**Up-regulated genes**
Xiong et al. (127)	IL-10, CCL2/MCP-1, CCR2, CXCL10/IP-10, CCL3/MIP-1A, CCR5, and CCL4/MIP1B
Liao et al. (128)	FCN1^hi^; FCN1^lo^SPP1^+^; FCN1^−^SPP1 inflammatory monocytes/macrophages in severe disease CD8 activation and effector molecules and higher CD8 TCR repertoire in moderate illness
Zhou et al. (129)	CXCL17, CXCL8 and CXCL2, CXCR2, CCL2, CCL7 IL-1β, ISGs, IL-17, TNF, and NF-κB signaling pathways

In another study, Liao et al. (128) used scRNA-seq and scTCR-seq to determine BALF cells' transcriptional signature in three patients with severe and another three with moderate COVID-19, and compared them with eight healthy subjects, previously studied. Their main findings were related to macrophages and CD8 cells. Macrophages were predominant in BALFs from patients with severe infection, with a minor proportion of T and NK cells, as compared with patients with moderate disease. Macrophages were classified in 22 clusters, according to their expression of *FCN1* (monocyte-derived), *SPP1* (pro-fibrotic), and *FABP4* (alveolar macrophages). These genes were differentially expressed both among the two groups of patients and the healthy controls. *FABP4* was preferentially expressed in healthy controls and in patients with moderate COVID-19, while *FCN1* and *SPP1* were expressed in patients with severe COVID-19. Further macrophages classification resulted in four groups: Group 1, FCN1^hi^ only; Group 2, FCN1^lo^SPP1^+^; Group 3, FCN1^−^SPP1^+^; and Group 4, FABP4^+^. Group 1 macrophages expressed genes associated with inflammatory monocytes; Group 2 expressed chemokines and interferon stimulated genes (ISG); Group 3, genes related with immune regulation and profibrotic events; and Group 4 were alveolar macrophage typical genes. According to the investigators, these results suggest that during SARS-CoV-2 infection, inflammatory monocytes (*FCN1*^+^) are recruited from the circulation into the lungs, where they differentiate into *SPP1*^+^ macrophages, constituents of the severe inflammatory reaction. Analysis of the BALF transcriptome showed that T and NK cells are increased in COVID-19 patients, compared to healthy controls, which according to their gene expression can be classified in NK, CD8, CD4, Tregs, and proliferating cells. An important finding was that genes related to activating molecules, migration, calcium signaling, and effector molecules were highly expressed by CD8 cells in patients with moderate infection, compared with patients with severe COVID-19; this further supports the role of CD8 cells in the elimination of the virus and their subsequent, protective immunity. In contrast, patients with severe disease had a higher expression of genes related to proliferation, energy generation, and initiation of translation. These results suggest that in patients with moderate infection CD8 cells are more differentiated and efficient, while in severe Infection T cells are in a proliferative stage. Additionally, the finding that the TCR repertoire is higher in CD8 than in CD4 cells, suggests a larger clonal expansion of the CD8 cells taking part in the resolution of the infection.

Zhou et al. (129) used metatranscriptomic sequencing to profile immune signatures in the BALF of eight COVID-19 patients, compared to 146 community-acquired pneumonia patients and 20 healthy controls. Their results show that in BALF from COVID-19, the differentially expressed genes (DEGs) included up-regulated proinflammatory chemokines genes, such as CXCL17, CXCL8, and CXCL2, as well as the CXCR2 receptor, critical to neutrophil recruitment, and CCL2 and CCL7, needed for monocyte recruitment. These authors also found that COVID-19 patients up-regulated IL-1β, antiviral Interferon stimulated genes (ISGs), and genes related to the IL-17, TNF, and NF-κB signaling pathways. In addition, the cellular analysis showed an increased neutrophil to lymphocyte ratio (NLR) in patients with COVID-19 compared to patients with other pneumonias.

Taken together, findings in BALF demonstrate both a highly dysregulated innate and adaptive immune response in the affected lungs of patients with COVID-19.

## Conclusions

Just 5 months after the initiation of the COVID-19 pandemic in China, which extended quickly worldwide to greatly impact public health and economies, the amount of information gathered on all aspects of the infection and the celerity with which the international scientific community has shared such information is truly amazing. However, given the haste to publish results, many manuscripts are in repositories, and still waiting for peer review. A note of caution is therefore in order, if such information is to be used in defining new diagnostic, therapeutic, or prophylactic protocols. It is also important to consider the brief amount of time elapsed since the beginning of the pandemic, during which time it has not been possible to gather sufficient results from *in vitro* and experimental animal models to ensure further understanding of COVID-19's biology. Even when considering these limitations, the information provided by the papers reviewed herein strongly supports quantitative and qualitative differences in the immune responses of those infected with SARS-CoV-2 which seem to correlate with the clinical manifestations of COVID-19. Although studies of asymptomatic infected individuals are lacking, the immunological profiles of patients with moderate infections indicate a protective T cell-dependent response, in contrast to patients with severe disease who exhibit an exacerbated systemic inflammation, with signs of T cells exhaustion.

The following fundamental aspects need to be defined through close collaboration between clinicians and basic researchers, with strong support from the public and private financial agencies:

1 The alterations of the immune regulation that allow the disease to advance from an asymptomatic or mild infection to a severe disease with poor prognosis. Translational immunological research focusing on the cellular and molecular aspects of the virus-host interaction, using sophisticated bioinformatics and system biology tools, must be pursued. This includes experimental animal models required for a deep understanding of COVID-19's immunopathogenesis. Besides patients with moderate and severe COVID-19, studies in humans must include seropositive asymptomatic individuals and patients with virologically confirmed mild infections. These subjects should be studied in long-term follow-up cohorts.
2 The genetics of resistance/susceptibility at the various stages of the infection and the disease. Topics like the resistance *per se* in exposed non-infected individuals, and the genetic risk factors for the progression from asymptomatic to moderate and severe disease must be prioritized. Initiatives like “COVID Human Genetic Effort” (www.covidhge.com) are working in that direction.
3 Based on the previous points it is necessary to find correlates of protective immunity and prognostic biomarkers to guide personalized management of infected individuals in order to prevent their progression to severe forms of the disease.
4 New pharmacological and immune-based treatments must be developed simultaneously with rigorous evaluation of treatments already available. The analysis of the currently available pharmacological treatments, or those under development, is beyond the scope of this review, but there is an excellent recent review about these treatments (130). Possible immunotherapies may include: convalescent plasma, already assayed in a small number of patients (131–133); monoclonal antibodies against the IL-6 receptor (112, 113) and interferon β (134, 135); and Leronlimab CCR5 blocking antibody (136), among others. Fortunately, a good number of controlled clinical assays have been initiated under strict supervision from regulatory agencies (https://clinicaltrials.gov/ct2/results?cond=covid&term=&cntry=&state=&city=&dist=) which will hopefully provide, within a prudential time, therapeutic agents for the efficient treatment of COVID-19 patients.
5 Development of vaccines to prevent, and hopefully eliminate, SARS-CoV-2 and other coronavirus infections. As expected, many investigators and biotechnology companies are dedicating all their efforts and resources to obtaining an effective vaccine in the shortest time possible. Although this topic is beyond the scope of this review, there are excellent reviews on the subject (29, 137) Worth mentioning are the different approaches, mostly targeting the S protein with its RBD. Vaccine candidates include RNA and DNA vaccines, recombinant proteins, and vectored vaccines, as well as inactivated and live attenuated vaccines. The first human trial published assessed the safety, tolerability, and immunogenicity of a recombinant adenovirus type-5 (Ad5) vectored expressing *S* protein of SARS-CoV-2 (138). One hundred ninety-five participants were allocated in three dose groups and followed for 28 days post-vaccination. Mild adverse reactions were common within the first 7 days after vaccination with no serious events noted during the observation period. Neutralizing antibodies were detected at Day 14 and peaked at Day 28 post-vaccination, and specific CD4 and CD8 cells peaked at Day 14 and remained present through Day 28 in the three dose groups. It is important to note that development of an efficient vaccine requires a deep understanding not only of the viral antigens and epitopes, but also of the immunological events leading up to the epitope presentation and recognition resulting in the establishment of a protective immune memory, the effector mechanisms in response to the antigens, and the adjuvants present in the proposed vaccine, one that would have minimal side effects (139).

Finally, it is important to remember what many investigators of SARS and MERS have written in their publications, long before the emergency of COVID-19 pandemics: what will be learned from this pandemic must be used to prevent future coronavirus epidemics.

## Author Contributions

LG: wrote and edited the manuscript.

## Conflict of Interest

The author declares that the research was conducted in the absence of any commercial or financial relationships that could be construed as a potential conflict of interest.
